# Deep RNA Sequencing Reveals Novel Cardiac Transcriptomic Signatures for Physiological and Pathological Hypertrophy

**DOI:** 10.1371/journal.pone.0035552

**Published:** 2012-04-16

**Authors:** Hong Ki Song, Seong-Eui Hong, Taeyong Kim, Do Han Kim

**Affiliations:** School of Life Sciences and Systems Biology Research Center, Gwangju Institute of Science and Technology (GIST), Gwangju, Republic of Korea; Kyushu Institute of Technology, Japan

## Abstract

Although both physiological hypertrophy (PHH) and pathological hypertrophy (PAH) of the heart have similar morphological appearances, only PAH leads to fatal heart failure. In the present study, we used RNA sequencing (RNA-Seq) to determine the transcriptomic signatures for both PHH and PAH. Approximately 13–20 million reads were obtained for both models, among which PAH showed more differentially expressed genes (DEGs) (2,041) than PHH (245). The expression of 417 genes was barely detectable in the normal heart but was suddenly activated in PAH. Among them, *Foxm1* and *Plk1* are of particular interest, since Ingenuity Pathway Analysis (IPA) using DEGs and upstream motif analysis showed that they are essential hub proteins that regulate the expression of downstream proteins associated with PAH. Meanwhile, 52 genes related to collagen, chemokines, and actin showed opposite expression patterns between PHH and PAH. MAZ-binding motifs were enriched in the upstream region of the participating genes. Alternative splicing (AS) of exon variants was also examined using RNA-Seq data for PAH and PHH. We found 317 and 196 exon inclusions and exon exclusions, respectively, for PAH, and 242 and 172 exon inclusions and exclusions, respectively for PHH. The AS pattern was mostly related to gains or losses of domains, changes in activity, and localization of the encoded proteins. The splicing variants of 8 genes (i.e., *Fhl1*, *Rcan1*, *Ndrg2*, *Synpo*, *Ttll1*, *Cxxc5*, *Egfl7*, and *Tmpo*) were experimentally confirmed. Multilateral pathway analysis showed that the patterns of quantitative (DEG) and qualitative (AS) changes differ depending on the type of pathway in PAH and PHH. One of the most significant changes in PHH is the severe downregulation of autoimmune pathways accompanied by significant AS. These findings revealed the unique transcriptomic signatures of PAH and PHH and also provided a more comprehensive understanding at both the quantitative and qualitative levels.

## Introduction

Cardiac hypertrophy involves multiple cellular signaling pathways and is a compensatory response to a variety of stimuli. While physiological hypertrophy (PHH) is associated with eccentric growth, pathological hypertrophy (PAH) is characterized by eccentric or concentric growth, depending on whether it occurs in response to pressure or volume overload [1]. Initially, PAH compensates for increased workload; however, its progression generally leads to detrimental remodeling and cardiac dysfunction [1]. In contrast, exercise training-induced PHH is a favorable response to physical activity; it is characterized by increased left ventricular volume and a proportional increase in wall thickness and mass [2]. However, the underlying molecular mechanisms responsible for the different types of hypertrophic adaptations remain to be elucidated.

Thus far, several genome-scale studies of cardiac hypertrophy have been conducted to determine comprehensive signatures by using the microarray method [3]–[6]. However, this method has several limitations such as spatial bias, uneven probe properties, low sensitivity, and dependency on the probes spotted [7]–[9]. Therefore, complete transcriptomic profiling of the different types of cardiac hypertrophy has not been performed. RNA sequencing (RNA-Seq) is a revolutionary alternative that can overcome the limitations of the microarray technique, enabling the sequencing of considerably large sequences in a short time. In addition, unlike microarrays, the RNA-Seq method has high reproducibility, sensitivity, and capability for capturing the splicing variants.

In this study, we performed a detailed RNA-Seq study to simultaneously elucidate both the quantitative and qualitative signatures of PAH and PHH. Initially, we conducted both RNA-Seq and microarray experiments to evaluate the efficacy of the RNA-Seq method in studies of mammalian hearts. We found that RNA-Seq provides considerably higher sensitivity, accuracy, and reproducibility than the microarray method for cardiac analysis. Therefore, the method of RNA-Seq was selected in the current study for examining cardiac transcriptomic signatures in the different hypertrophy models. Further system-level approaches using the differentially expressed genes (DEGs) derived from the PAH and PHH samples showed that the signaling pathways and the expression of the genes involved in PAH are strikingly different from those of PHH. Notable differential transcriptome changes were also observed in alternative splicing (AS) patterns in the hypertrophy models.

Taken together, in this RNA-Seq study, we showed distinct transcriptomic signatures related to PHH and PAH, which could provide useful insights into understanding the mechanisms underlying PHH and PAH.

## Results

### Animal models and high-throughput sequencing

In order to study the transcriptomic signatures of cardiac hypertrophy, we generated 2 different mouse models for cardiac hypertrophy. C57BL/6J mice were subjected to swim-training and pressure overload by trans-aortic constriction (TAC) to produce models of PHH and PAH, respectively. In both models, the hearts were significantly enlarged as evidenced by the heart weight to body weight (HW/BW) ratio (*p*<0.01) (Figure S1A). The extent of hypertrophy was determined by quantitative real-time PCR (qRT-PCR) for 5 hypertrophy markers: *Nppa*, *Nppb*, *Myh7*, *Acta1*, and *Pln*. Although the hypertrophied hearts had similar appearances, the expression levels of the markers significantly differed between the PHH and PAH mice. In PAH mice, *Nppa*, *Nppb*, *Myh7*, and *Acta1* were considerably upregulated, while *Pln* was downregulated (Figure S1B). However, in PHH mice, *Nppa* was upregulated, *Acta1* was downregulated, and the *Nppb*, *Myh7*, and *Pln* expression levels remained unchanged. The expression levels of these markers in PHH and PAH are comparable with those reported in previous studies [10]–[14].

**Figure 1:**
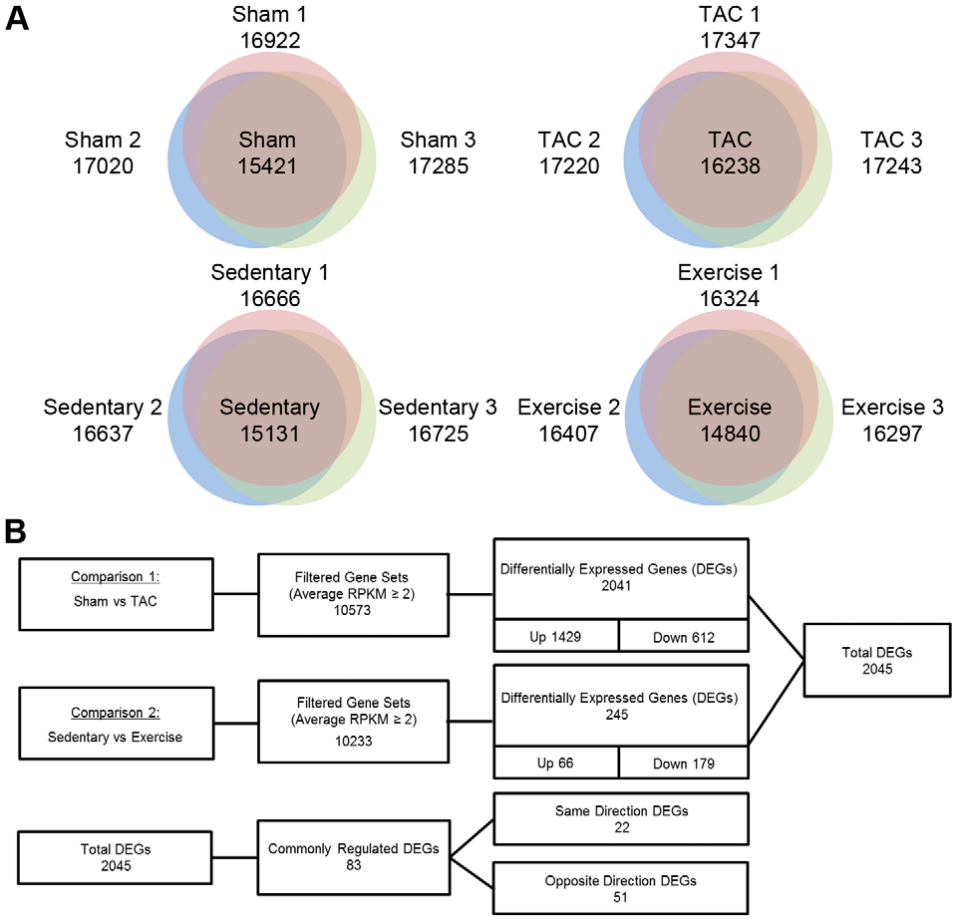
Summary of differentially expressed genes (DEGs) in pathological hypertrophy (PAH) and physiological hypertrophy (PHH). (A) Venn diagrams to show the number of expressed genes detected at least once for each biological replicate of sham, TAC, sedentary and exercise-training mouse models. (B) Comparisons of DEGs between the different mouse models. Genes having at least 2 RPKM value and differentially expressed at (*p*<0.05, |log_1.5_ fold change (FC)|≥1) were considered for comparisons.

For both models, we performed RNA-Seq by using an Illumina Genome Analyzer II (GA-II), and approximately 22–26 million reads were obtained. From these reads, low-quality reads were eliminated, resulting in 13–20 million reads (equal to 60–70% of the total reads). In total, 12–13 and 7–8 million reads were aligned to the mouse genome (mm9) for PAH and PHH, respectively (Table S1). The numbers of reads for genes were further normalized to “reads per kilobase of exon model per million mapped reads” (RPKM); thus, the values were considered the final expression levels for each gene [15]. According to Mortazavi et al. [15], a single gene copy is equivalent to 1∼3 RPKM, depending on the cell type [15]. We also observed at least 2 RPKM genes expressed in the heart (i.e., *Bcl2*, *Myc*, *Pex1*, and *Eya2*). Therefore, we discarded the genes with fewer than 2 RPKM to reduce false positives. Finally, the analysis showed that 10,233–10,573 genes were expressed in the hearts (Figure 1). The gene expression profiles derived from RNA-Seq were further compared to the profiles by using the microarray technique. To efficiently compare the techniques, the RPKM values of genes were converted into the logarithmic value of “reads per kilobase of exon model per billion mapped reads” (RPKB). Although highly consistent profiles were obtained for each profiling method (R^2^∼0.96–0.98), the correlations between techniques were significantly lower at approximately 0.62, indicating technological differences (Figure S2). As shown in Figure S3, RNA-Seq exhibited much higher sensitivity than the microarray technique, as evidenced by the significantly increased number of DEGs (n = 2,041 vs. 891). To verify the accuracy of the 2 techniques, we performed qRT-PCR for 51 genes. The expression patterns were consistent for RNA-Seq and qRT-PCR (R^2^ = 0.86–0.89), in contrast with the results for the microarray method (R^2^ = 0.67). These results suggest that RNA-Seq consistently provides considerably higher sensitivity and accuracy than the microarray method [16], [17].

### DEGs and network analysis

**Figure 2:**
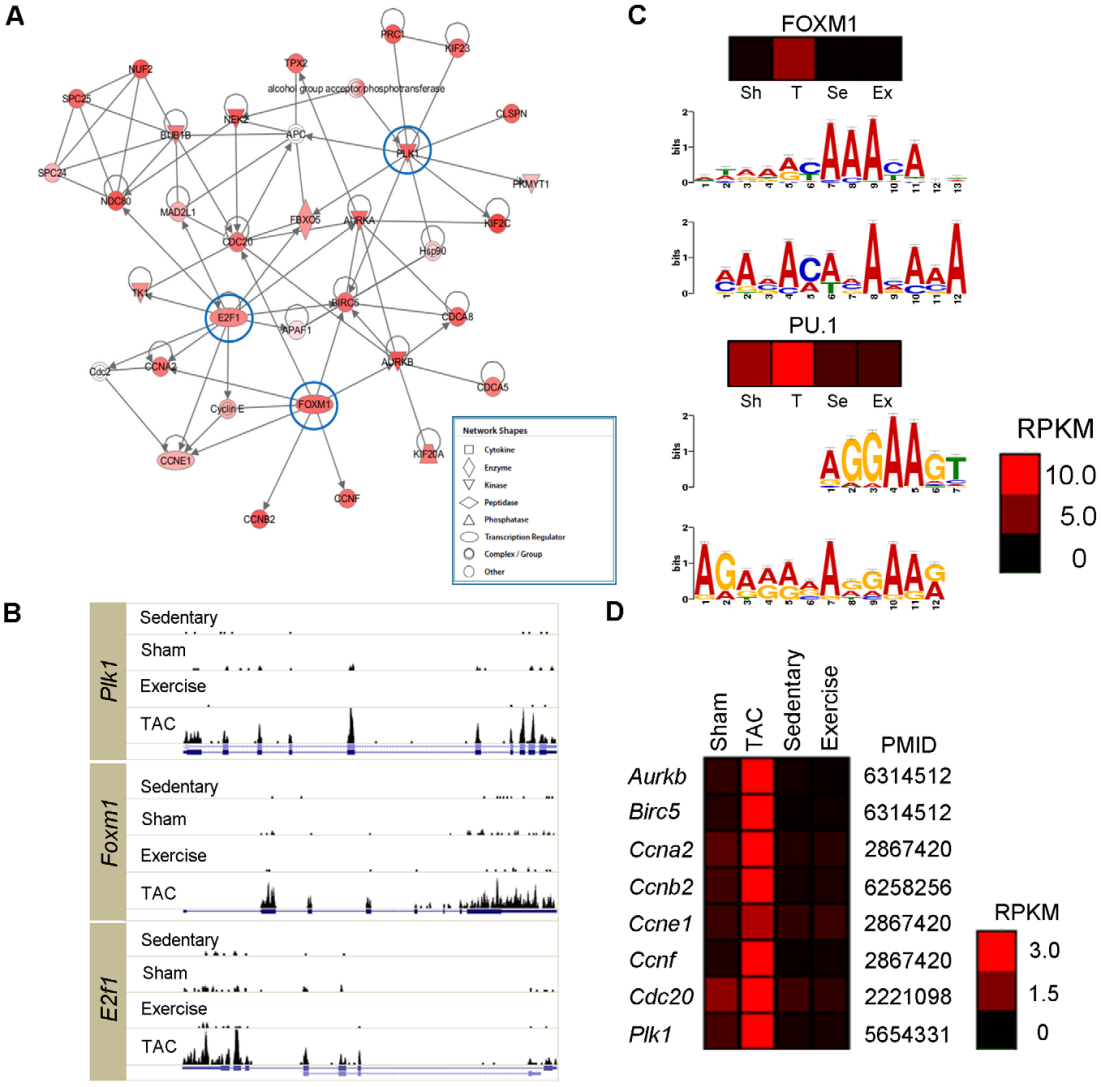
Network of the genes turned on in PAH. (A) Top-scoring network derived from the 417 genes dramatically increased in PAH, despite their extremely low expression (<2 RPKM) in either normal adult heart or exercise-trained heart. The red-colored nodes represent up-regulated genes. (B) The histograms for the reads that were mapped by the UCSC isoforms of *Plk1*, *Foxm1* and *E2f1* in the different animal models. The blue lines underneath represent UCSC gene structures and the boxes show the exons of the genes. Expression of the 3 genes is only dominant in TAC. (C) The predicted motifs enriched in 1,000 bp upstream of 417 genes. The motifs shown on the bottom were predicted by MEME and the motifs shown above are the consensus motifs for the TFs (FOXM1 and PU.1). Y-axis indicates the amount of information at each position in the motif. The heatmaps (unit: RPKM) show the expression levels of the predicted TFs in sham (Sh), TAC (T), Se (Sedentary) and E (exercise). (D) Degree of expression (unit: RPKM) of the known targets of FOXM1 in the animal models are shown along with the previous evidence (PMID).

DEGs associated with PAH and PHH were analyzed using the following criteria: *p*<0.05 (Student's *t*-test), |fold change|≥1.5, and expression level ≥2 RPKM. The analysis showed 2,041 and 245 DEGs for PAH and PHH, respectively (Figure 1B). In addition to the striking difference in the number of DEGs between the 2 types of hypertrophy, the DEG patterns were clearly different. For instance, 70% of all DEGs (1,429 genes) were upregulated in PAH, whereas 73% were downregulated in PHH (179 genes). Interestingly, the expression of 417 genes (29%) was barely detectable in normal hearts (i.e., RPKM<2) but considerably increased in PAH. The remarkable features of these genes were determined from the gene networks inferred by Ingenuity Pathway Analysis (IPA, http://www.ingenuity.com). According to the top-scoring network, the abundant genes were cell cycle regulators such as E2F transcription factor 1 (*E2f1*), forkhead box M1 (*Foxm1*) and polo-like kinase 1 (*Plk1*) (Figure 2A). The expression levels of *E2f1*, *Foxm1* and *Plk1* in PAH were clearly distinguishable from those in PHH, as shown in Figure 2B. While the basal expressions of *E2f1*, *Foxm1* and *Plk1* were extremely low (RPKM<2), the expressions of the genes were 5.3∼8.9 times greater in PAH.

We further analyzed the 1000 bp upstream sequence of these 417 genes to identify transcription factor (TF) binding motifs using MEME [18]. The putative binding motifs for FOXM1 (*p* = 1.9×10^−5^) and PU.1 (*p* = 4.8×10^−21^) were significantly enriched in the 1,000 bp upstream of the genes encoding cell cycle regulators (Figure 2C). The strong expression of FOXM1 (7.7 fold greater in PAH than sham) and the known downstream targets (Figure 2D) suggest that it plays a critical role in pathogenesis. FOXM1 is known to be important for cardiac development [19], however, there is no report regarding the role of FOXM1 in cardiac hypertrophy. The binding motif for PU.1 was also highly enriched in the upstream region. The expression of *Sfpi1* encoding PU.1 remained moderate in the basal state but increased significantly by 2.33 fold (14.46 RPKM) in PAH, suggesting its involvement in pathogenesis. Although Wontakal et al. recently reported that PU.1 ChIP-Seq peaks are abundant within the upstream regions of cell cycle regulators in erythroid cells [20], the specific roles of PU.1 in the cell cycle or cardiac hypertrophy remain unknown. Highly conserved motifs for PITX2 and PAX were identified, but the expression levels of *Pitx2* and *Pax* encoding PITX2 and PAX, respectively, were extremely low in PAH (data not shown).

We further analyzed 52 genes that were oppositely regulated in the 2 hypertrophy models. The top-scoring networks for 52 genes were highly enriched with genes encoding collagens, chemokines, and actin (Figure S4A). To predict the master TFs for 52 genes, we analyzed the enriched motifs within 1,000 bp upstream of the genes. We identified the strong enrichment of two MAZ consensus motifs, GGGAGGG (*p* = 1.0×10^−10^) and CCCTCCC [21], [22] (Figure S4B). According to a recent finding, MAZ may regulate the expression of muscle specific genes via these consensus motifs [23]. Taken together, our study suggests that MAZ is an important TF for both types of cardiac hypertrophy.

### Analysis of exon variants

AS is an important regulatory mechanism in gene expression; it generates considerable versatility at the post-transcriptional level and accounts for proteome complexity. Recent studies have estimated that 75–92% of human genes undergo AS events [24], [25]. In the heart, several genes (e.g., *Tnnt2*, *Camk2d*, *Ncam*, and *Pdlim5*) are reported to undergo AS during cardiac development [26]–[29]. However, few AS events have been reported in adult hearts to date. In the present study, we analyzed exon inclusions/exclusions in terms of known alternative exon variants on the basis of a UCSC “knownAlt” (Fisher's exact test, *p*<0.05; Bayesian error rate, *e*≤0.1). As shown in Figure S5A, we identified 317 and 196 exon inclusions and exclusions, respectively, in PAH, and 242 and 172 exon inclusion, respectively, in PHH. Alternative promoter usage (43% and 37% for PAH and PHH, respectively) followed by cassette exons (26% and 24% for PAH and PHH, respectively) were the most abundant types of AS events (Figure S5B, C).

To determine which functions are affected by AS events, we searched previous in-depth studies on exon variants. According to the previous studies (Table 1), the biological properties altered by AS were classified into the following categories: (1) domain gain or loss, (2) activity change, and (3) localization. The most abundant feature was gains or losses of domains, as observed in *Limk2*, *Enah*, *Sox17*, *Aak1*, *Itsn1*, and *Plec1*. The Lim, PDZ, and clathrin-binding domains disappeared in *Limk2t* and *Aak1s* during PAH [30], [31]. The domain variants for EPS15 homology, Src homology, and actin-binding domains were observed in *Itsn1* and *Plec1* during PAH. However, the exon encoding the high-motility group (HMG) domain of *Sox17* was included in PHH (Table 1). Differential activity or sensitivity was predicted in *Cav1α*, *Pde1c*, and *Ncoa1* on PI3K/Akt (for *Cav1α*), Ca^2+^, cAMP, cGMP (for *Pde1c*), and thyroid hormone (for *Ncoa1*) [32]–[34]. In addition, AS influenced localization in *Sorbs1*, *Ehmt2*, and *Plec1* (Table 1) [35]–[37].

**Table 1:** List of genes and isoforms derived from different alternative splicing variants in PAH and PHH.

					Exclusion/Inclusion			Isoform	
Gene	Exon position	AS type	Function	Ref (PMID)	PAH	PHH	Modification	Name	Prevailing type in hypertrophy
*Aak1*	chr6:86946958-86953221	altFinish	protein amino acid phosphorylation, etc	17494869	Exclusion		D	AAK1L, AAK1S	AAK1S for PAH
*Ablim2*	chr5:36209354-36209415	cassetteExon	regulation of transcription, etc	16005990	Exclusion			Ablim2 a/b	Ablim2b
*Bsg*	chr10:79171442-79171789	cassetteExon	pyruvate metabolic process, etc	18434307	Exclusion	Inclusion		Bsg1, Bsg2, Bsg3, Bsg4	Bsg1 for PHH
*Cacna2d2*	chr9:107429478-107429567	strangeSplice	transport of calcium ion, etc	11130987	Inclusion				
*Cav1*	chr6:17256370-17256479	altPromoter	endocytosis, cholesterol transport, etc	15067006	Inclusion		A	Cav1 α/β	Cav1α
*Cdk9*	chr2:32568085-32568305	altPromoter	regulation of transcription, etc	12706900		Exclusion		CDK9_42_/CDK9_55_	CDK9_42_
*Ddx5*	chr11:106645415-106645476	altPromoter	RNA splicing, cell growth, etc	10648785		Exclusion		2.3 kb vs. 4.4 kb	
*Dsc2*	chr18:20189299-20190904	bleedingExon	cell adhesion, etc	14673151	Exclusion				
*Egfl7*	chr2:26436603-26436761	altPromoter	angiogenesis, multicellular organismal development, negative regulation of cell migration, etc	14592969	Inclusion	Exclusion		VE-statin-a, VE-statin-b	VE-statin-b for PHH, VE-statin-a for PAH
*Ehmt2*	chr17:35048308-35048423	cassetteExon	regulation of transcription and M-phage, etc	11707778	Inclusion		L	NG36/G9a, NG36/G9a-SPI	
*Enah*	chr1:183851789-183852631	altThreePrime	actin remodeling, etc	20565797	Inclusion	Inclusion	D	Enah/Evl/Vasp	Enah
*Gata4*	chr14:63863988-63864097	altPromoter	regulation of cardiac muscle cell proliferation, etc	20041118	Inclusion				
*Gtf2i*	chr5:134790253-134790616	altPromoter	regulation of transcription, etc	19111598	Inclusion				
*Gtf2ird1*	chr5:134838381-134838418	cassetteExon	regulation of transcription, etc	12780350	Exclusion			BENα, BENβ, BENγ	BENγ
*Itsn1*	chr16:91861439-91861484	cassetteExon	apoptosis, etc	19777371	Exclusion		D	Itsn1 short form 1∼14, long form 1∼4	short form 3/6/7/10/11/13/14 or long form 4
*Kcnip2*	chr19:45871581-45871676	cassetteExon	ion transport, muscle contraction, etc	16112838	Exclusion				
*Limk2*	chr11:3271345-3271495	altPromoter	actin cytoskeleton reorganization, etc	9610354	Inclusion		D	Limk2 a/t	Limk2t
*Mapk14*	chr17:28828390-28828800	altPromoter	myocyte cell death and glucose metabolism process,etc	16507160	Inclusion	Inclusion		Mxi2, p38, CSBP1	
*Mxi1*	chr19:53403903-53404210	altPromoter	negative regulation of cell proliferation, transcription regulator activity, etc	19254710	Exclusion			Mxi1-0, Mxi1-1	Mxi1-0 for PAH
*Ncoa1*	chr12:4249836-4249890	cassetteExon	proliferation, apoptosis, etc	9223431		Exclusion	A	SRC1, SRC1(Q), SRC1E	SRC1/SRC1(Q) for PHH
*Ndrg2*	chr14:52530761-52530802	cassetteExon	cell growth, cell differentiation, etc	17688410	Exclusion			Ndrg2a1, Ndrg2a2, Ndrg2b1, Ndrg2b2	Ndrg2b2
*Nfix*	chr8:87247627-87247749	cassetteExon	DNA replication, etc	20098426	Inclusion	Exclusion			
*Pcyt2*	chr11:120474344-120474397	cassetteExon	phospholipid biosynthetic process, biosynthetic process, etc	14697519	Exclusion	Exclusion		Pcyt2α, Pcyt2β	Pcyt2β for both
*Pde1c*	chr6:56295120-56295146	cassetteExon	nucleotide dephosphorylation, etc	8810348	Inclusion	Exclusion	A	Pde1c1/2	Pde1c2 for PHH, Pde1c1 for PAH
*Plec1*	chr15:76025854-76026140	altPromoter	actin cytoskeleton dynamics, etc	14559777	Inclusion		D, L		
*Rorc*	chr3:94176708-94176853	altPromoter	regulation of transcription, etc	9403063	Inclusion				
*Scarb1*	chr5:125761475-125761603	cassetteExon	cell adhesion, positive regulation of nitric-oxide synthase activity, etc	20085651	Inclusion			SR-BI, SR-BII	SR-BI for PAH
*Smox*	chr2:131347795-131347955	altThreePrime	polyamine and xenobiotic metabolic process,oxidation reduction, etc	12398765		Exclusion		PAO1∼4	PAO3 for PHH
*Sorbs1*	chr19:40451135-40451506	altFivePrime	cytoskeleton rearrangement, etc	12765336	Inclusion		L	CAP1∼4	CAP4
*Sox17*	chr1:4483181-4483547	cassetteExon	regulation of transcription, etc	8636240		Inclusion	D	Sox17, t-Sox17	Sox17 for PHH
*Spag9*	chr11:93984036-93987397	retainedIntron	spermatogenesis and migration, etc	19056739		Exclusion		Spag9/Jip-4	
*Tacc2*	chr7:137765235-137770355	cassetteExon	microtubule cytoskeleton organization regulation of microtubule-based process, etc	12620397	Inclusion			Tacc2l, Tacc2s	Tacc2l
*Tmpo*	chr10:90616023-90616142	cassetteExon	regulation of transcription, etc	8743987	Inclusion	Exclusion		Tmpo α, β, β′, γ, ε, δ, ζ	Tmpo β for PAH

Modification type: D, gain or loss of domain; A, activity; L, localization.

### Experimental confirmation of exon variants

**Figure 3:**
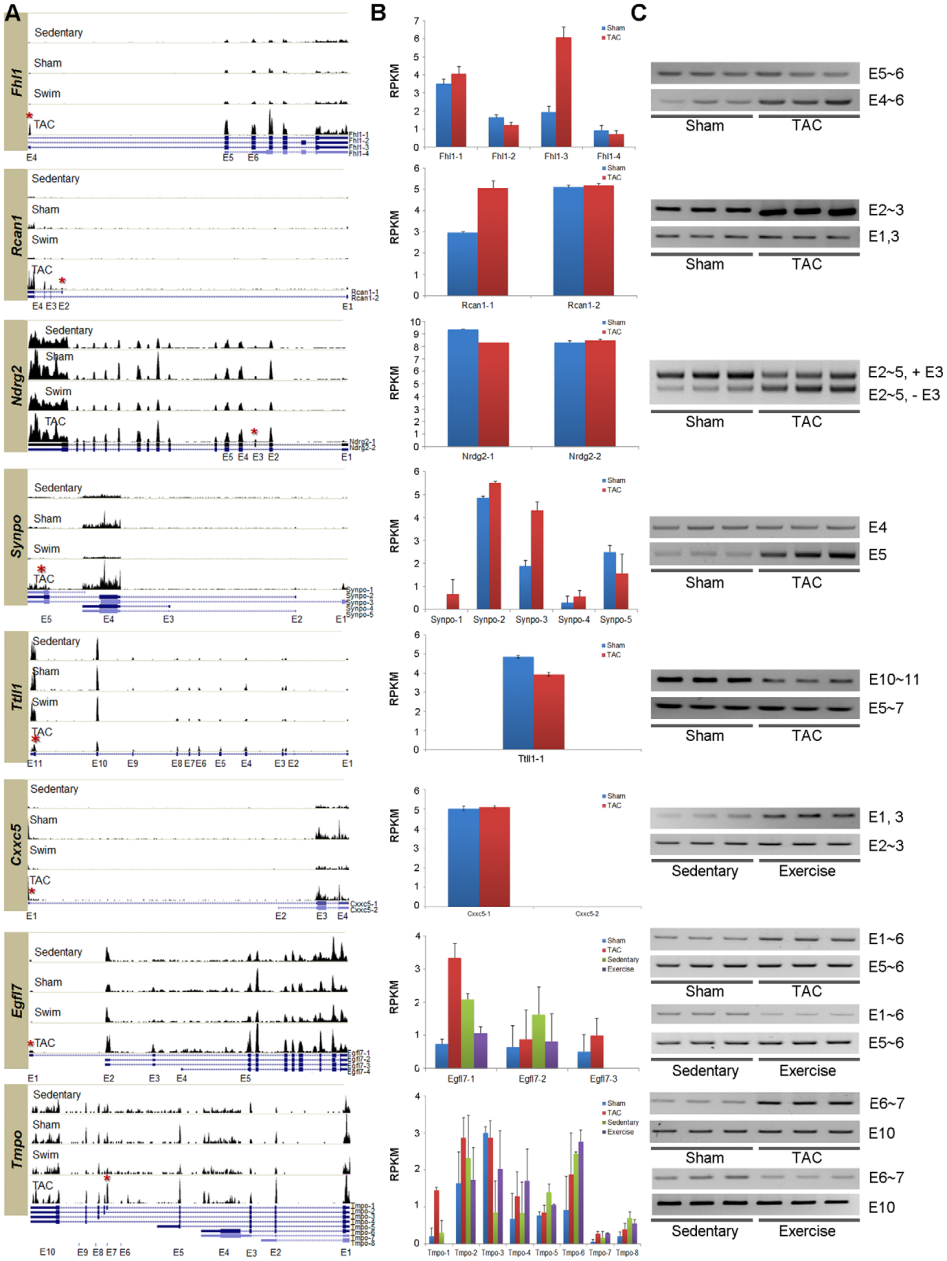
Experimentally verified isoforms alternatively spliced in PAH and PHH. (A) The histograms of mapped reads for 8 genes. The red asterisks shown at UCSC isoforms (blue lines underneath) indicate the exons (shown as blue boxes) alternatively spliced in hypertrophic signal-specific manners. The degree of expression is shown as vertical length. (B) Distribution of UCSC isoforms for the matching 8 genes characterized in different hypertrophy models. Different distribution of isoforms in either PAH or PHH was analyzed using NEUMA. The average RPKM values are shown with standard errors (N = 3). (C) Experimental confirmation of exon variants for the matching 8 genes using RT-PCR. For each RT-PCR results, the amplified regions with the specific primers are described. The detailed information for the primers used for the detection of the exons is available in Table S2. *Fhl1*, *Rcan1*, *Ndrg2*, *Synpo* and *Ttll1* were experimentally confirmed in PAH and the splicing pattern of *Cxxc5* exon variants was confirmed in PHH. For *Egfl7* and *Tmpo*, the opposite splicing patterns were confirmed for both PAH and PHH. The isoform distributions of *Ttll1* and *Cxxc5* were not analyzed, since the UCSC isoforms for *Ttll1* is not reported and there were no mappable reads for *Cxxc5-2*. The exon numbers amplified are indicated on the right. For instance, E5∼6 means exon 5 and 6 were amplified with specific primers shown in Table S2. For *Ndrg2*, two isoforms (E2∼5, +E3 and E2∼5, −E3) were shown in the same gel.

To validate the exon variants, we designed RT-PCR assays. 10 exon variants for 8 genes were selected for validation. The expression of the exon variants and their constitutive exons was compared. The results confirmed that the exon variants corroborated the RT-PCR results (Figure 3). To confirm the exon variants associated with the different types of cardiac hypertrophy, the isoform analysis was performed using NEUMA [38]. For the *Fhl1* gene, both quantitative and qualitative changes were simultaneously observed, i.e., its expression increased by 3.5 fold, and the *Fhl1-3* isoform specifically increased during PAH. Although the most abundant isoform was *Fhl1-1*, *Fhl1-3*, which encoded additional 16 amino acids, increased with alternative promoter usage. Similarly, alternative promoter usage was also observed in *Rcan1* isoforms. Along with the strong 2.97-fold increase, the proximal promoter usage resulted in specific increase of *Rcan1-1*, which produced slightly different N-terminal sequences. In the case of *Ndrg2*, differential cassette exon usage was observed in PAH. Among the 2 isoforms of *Ndrg2*, the shorter isoform excluding the exon at the 52,530,761∼52,530,802 region of chromosome 14, was significantly upregulated in PAH. Quantitative reduction of ∼30% was found in *Ndrg2*, suggesting that the expression reduction is mostly found in *Ndrg2-1*. For the isoforms of *Synpo*, relatively strong expression of the exon positioning at 60,753,627∼60,756,142 region of chromosome 18 was observed, contrary to the constant expression of the previous exon (i.e., E4). The isoform analysis showed that the most significantly increased isoform was *Synpo-3*. However, specific activity of *Synpo-3*, which increased significantly in PAH, has not yet been reported.

Although no isoform for *Ttll1* has been reported in the UCSC mouse genome database, we observed that the last exon of *Ttll1* was strongly excluded, indicating the existence of isoforms. The histograms for the reads mapped into *Ttll1* and the significant decrease in the expression of exons 10∼11, as shown by RT-PCR, also supported the possibility that *Ttll1* has multiple isoforms in PAH. We also identified the exon variants regulated in PHH. *Cxxc5* underwent an AS event in PHH, specifically alternative promoter usage for the gene. Among the 2 *Cxxc5* isoforms derived from the alternative promoters, the transcript from the distal promoter increased significantly during exercise, in contrast to the constant expression of the transcript with the proximal promoter. These *Cxxc5* isoforms are likely to produce the same proteins even with different 5′ UTRs, implying the possibility of post-transcriptional regulation. Among the exon variants, hypertrophic signal-specific regulation was apparent in *Egfl7* and *Tmpo*. According to the UCSC genome browser analysis, 3 different alternative promoters were involved in the AS for *Egfl7*. Significantly increased usage of the most distal promoter (i.e., *Egfl7-1*) was confirmed in PAH by RT-PCR; the usage of the promoter decreased in PHH [39]. Signal-dependent splicing regulation was also found in *Tmpo*. The inclusion of exon 6 in PAH was experimentally confirmed by RT-PCR, suggesting the relatively increased proportion of *Tmpo-1* encoding TMPOβ [40]. The opposite splicing pattern (i.e., exclusion) was found in PHH. The opposite regulation was also found at the quantitative level, i.e., the expression decreased slightly and increased in PHH and PAH, respectively. These findings suggest multilateral regulation both in quantitatively and qualitatively according to the hypertrophic signals.

### Critical pathways involving DEGs and splicing variants

**Figure 4:**
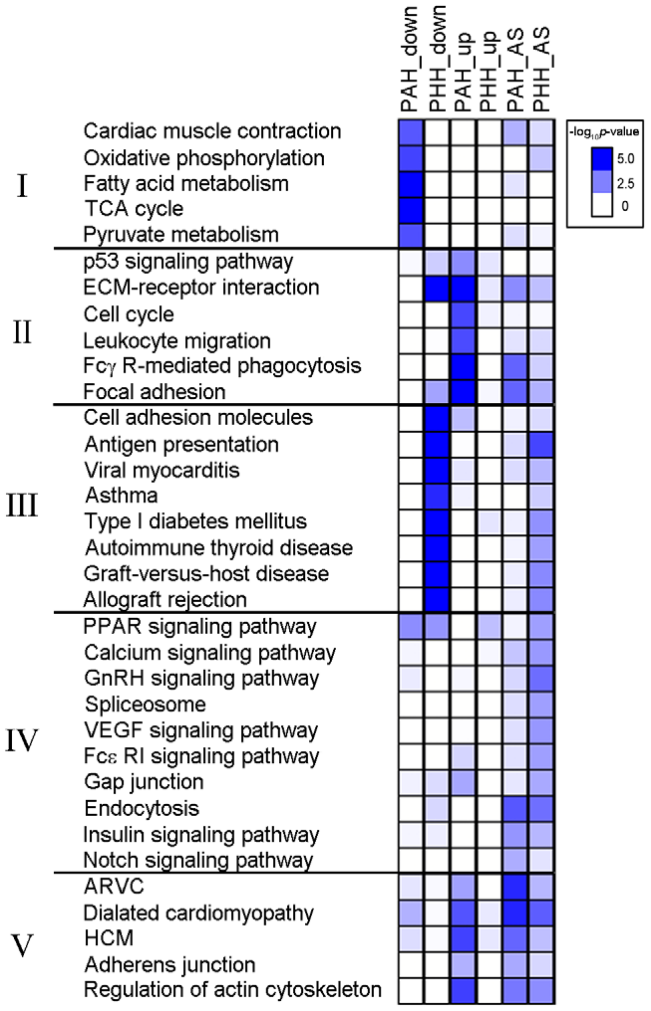
Critical pathways associated with DEGs and exon variants changed in PAH or PHH. Significantly enriched pathways (*p*<0.05) with at least 5 DEGs or 5 exon variants for PAH and PHH are shown. Color intensity represents degree of enrichment (−log_10_[*p*-value]). The significant pathways were categorized. (Group I) pathways for muscle contraction and metabolism. (Group II) pathways mainly for immune and cell cycle. (Group III) pathways for autoimmunity. (Group IV) pathways for cell signaling. (Group V) pathways mainly for cardiac diseases.

The critical pathways associated with DEGs and splicing variants were investigated using 194 KEGG pathways [41]. The pathways enriched with the up- or downregulated DEGs and splice variants for PAH and PHH are shown in Figure 4.

The enrichment patterns for the pathways could be categorized into 5 groups. Group I involved pathways for muscle contraction and metabolism. Clear transcriptomic signatures were observed in the genes involved in cardiac muscle contraction and various metabolic processes (e.g., oxidative phosphorylation, fatty acid metabolism, the TCA cycle, and pyruvate metabolism). The genes were significantly downregulated only in PAH, with little AS. However, there were neither quantitative nor qualitative changes in PHH. Group II mainly involved pathways for immune function and cell cycle: The common feature among p53 signaling, ECM, cell cycle, leukocyte migration, Fcγ R-mediated phagocytosis and focal adhesion was the strong enrichment of up-regulated genes in PAH. Among these, the quantitative changes in the genes involved in ECM, Fcγ-mediated phagocytosis and focal adhesion were combined with the AS in PAH. The most dynamic regulation was observed in ECM in that the gene exhibited distinguishable expression patterns between PAH and PHH, along with considerable AS in PAH. Group III mainly involved pathways implicated in autoimmunity. In PHH, the most striking features were the remarkable suppression of autoimmune systems such as cell adhesion, antigen presentation, viral myocarditis, allograft rejection, and autoimmune thyroid diseases. In PHH, the autoimmunity genes were significantly downregulated and active AS was seen. However, the same changes were not observed in PAH. Transcriptomic suppression and AS were observed in the major histocompatibility complex (MHC) in PHH. Group IV involved pathways for cell signaling. It is notable that in PHH, the major regulatory mechanisms for various signaling pathways, splicesomes and gap junctions exhibited qualitative remodeling via AS. For these pathways, quantitative changes were hardly detected in PHH. In the case of endocytosis, remarkable qualitative regulation was observed in both PAH and PHH, whereas there was no significant quantitative change in either cardiac hypertrophic model. Interestingly, the splicing patterns and targets involved in endocytosis were unique, depending on the hypertrophic signals. For example, exon 6 of *Clta* was a common splicing target; it was excluded in PAH but included in PHH. Group V mainly involved pathways related to cardiac diseases. Pathological signatures were apparent in PAH with quantitative and qualitative enrichment of pathways related to cardiovascular diseases such as hypertrophic cardiomyopathy (HCM), arrhythmogenic right ventricular cardiomyopathy (ARVC), and dilated cardiomyopathy (DCM). For PHH, the significant changes via AS were apparent in DCM and the actin cytoskeleton, suggesting that these pathways are the critical targets for regulation as a result of hypertrophic signals.

## Discussion

While the enlargement of the heart in PHH and PAH appears similar at the morphological level, the outcomes of the 2 types of hypertrophy are strikingly different. PHH leads to a considerable improvement in quality of life through increased oxidative capacity and maximal cardiac output, whereas PAH is associated with progressive deterioration of heart functions, ultimately leading to fatal heart failure. Therefore, characterizing both PHH and PAH at the molecular and systemic levels is essential for identifying the critical signaling pathways leading to adaptation or mal-adaptation. Thus far, the detailed mechanisms underlying the progression of hypertrophic processes have not been elucidated. In the present study, we used the RNA-Seq technique to profile near-complete transcriptomes in both PHH and PAH.

The novel findings of this study are as follows: (1) The number of DEGs was considerably higher in PAH than in PHH (8-folds higher) (Figure 1). (2) We found that 417 silent genes were turned on in PAH, whereas the expression of 52 genes was oppositely regulated between PAH and PHH (Figure 1). (3) FOXM1 was identified as a putative transcription factor involved in the pathogenesis of PAH (Figure 2). (4) The 52 genes differentially regulated between PAH and PHH are related to collagen, actin, and chemokine and MAZ was identified as a putative TF for the participating genes (Figure S4). (5) Substantial AS (both exon inclusion and exclusion) leading to a gain or loss of domains as well as changes in the activity and localization of the participating proteins were identified from RNA-Seq data for both PAH and PHH (Figure 3 and Table 1). (6) According to the multilateral pathway analysis using RNA-Seq data, 5 different patterns distinguishing PAH and PHH were found (Figure 4): group I (includes pathways for muscle contraction and metabolism), downregulated DEGs with mild AS in PAH; group II (mainly involving pathways for immunity and cell cycle), upregulated DEGs with considerable AS in PAH; group III (mainly involving pathways implicated in autoimmunity), downregulated DEGs with considerable AS in PHH; group IV (involving pathways for cell signaling), considerable AS in PHH; and group V (mainly involving pathways for cardiac diseases), upregulated DEGs with considerable AS in PAH.

### RNA-Seq vs. microarray

In the present study, we used RNA-Seq and microarray methods in order to understand the transcriptomic signatures for cardiac hypertrophy. The major advantages of RNA-Seq over microarrays are its significantly improved detection accuracy, ability to identify AS without probe dependency, and *de novo* analysis of novel transcripts and long non-coding RNAs [42]. RNA-Seq also exhibited a considerably greater capacity to identify DEGs (>40%), with considerably higher accuracy than microarrays (Figure 1). This critical advantage helped us expand our understanding, as exemplified by the significant enrichment of the gap junction pathway in PAH. In the gap junction pathway, 3.3-fold more DEGs (n = 20) were observed with RNA-Seq than with microarrays (n = 6). Our prior knowledge regarding the involvement of the gap junction pathway in cardiac hypertrophy further indicates that RNA-Seq identifies DEGs much more accurately.

However, RNA-Seq has some limitations, including limited software, bioinformatic algorithms, and standardization. For example, the rational criteria defining gene expression have not yet been standardized; therefore, the cutoff values for determining background expression levels vary widely across studies (e.g., 95% confidence level and >100 read count). Such variations cause differences in results. Therefore, a sophisticated algorithm for determining gene expression is a challenging issue that needs to be addressed. In certain cases including some histone genes the microarray method performed better than RNA-Seq. Genes that were not detected by RNA-Seq exhibited common characteristics, for example no polyadenylation in eukaryotes. These results are due to the procedure for mRNA isolation, which involved oligo-dT magnetic beads. Therefore, it is necessary to be careful when comparing RNA-Seq with microarrays. Since no gene annotation data in mouse mitochondria were present in the UCSC database, we excluded the RNA-Seq reads of the mitochondrial genome. From the RNA-Seq reads aligned to the mitochondrial chromosome, 13–15% of RNA-Seq reads were mapped to the mitochondrial chromosome in sham, sedentary, and swim-trained animal models; however only 9% of reads were mapped to the mitochondrial chromosome in the TAC model mice (data not shown).

### Top-scoring Ingenuity networks for identifying critical molecules involved in hypertrophy

The top-scoring Ingenuity network suggests that *Foxm1* plays an important role in the pathogenesis of PAH (Figure 2). Although *Foxm1* is known to be involved in tumorigenesis [43], [44] and cardiac development [19], its role in cardiac hypertrophy remained unknown until now. Our prediction of *Foxm1* as an important TF for PAH is based on the remarkable sensitivity of RNA-Seq as well as critical analysis of the network involved (Figure 1). The mechanism leading to PAH by *Foxm1* must be related to cell cycle regulators such as *Plk1* and *E2F1*. The tight regulatory loops among genes, including *Foxm1* and *Plk1*, have already been identified in other animal models [45]. The results also suggest a regulatory role of *Sfpi1* encoding PU.1in the *Foxm1*-*Plk1* loop, since the putative binding motifs of PU.1 were found upstream of these genes. In contrast to the results for *Foxm1* and *Plk1*, *Sfpi1* is expressed in the adult heart, and its expression is significantly elevated in PAH. The functional role of *Sfpi1* in cell cycle regulators has not been studied thus far, although its binding targets were identified by Chip-Seq experiments [20].

We further analyzed 52 genes oppositely regulated in the 2 hypertrophy models (Figure S4). The network for these genes was highly correlated with collagen, chemokine, and actin genes. To predict the master TFs for 52 genes, we examined the enriched motifs within 1,000 bp upstream of the genes and identified the strong enrichment of the MAZ consensus motif, GGGAGGG (*p* = 1.0×10^−10^) and CCCTCCC (*p* = 6.6×10^−11^) [23]; this result supports the potential role of MAZ in the regulation of those genes. Collectively, our study results predicted several critical genes that may be involved in the pathogenesis of hypertrophy. However, further molecular characterizations are required in the future.

### Examples of alternative splicing in PAH and PHH

Compared with microarray, RNA-Seq could provide a great advantage by enabling the simultaneous analysis of both DEG and AS without platform dependency. With the aid of RNA-Seq, we identified 470 and 387 genes containing known alternative exons in PHH and PAH, respectively. Of these, we experimentally confirmed AS for 8 genes, i.e., *Fhl1*, *Rcan1*, *Ndrg2*, *Synpo*, *Ttll1*, *Cxxc5*, *Egfl7*, and *Tmpo* (Figure 3). Among them, *Fhl1* exhibited complicated expressional regulation, both quantitatively and qualitatively. The *Fhl1* gene encodes 3 different isoforms (*Fhl1*, *Fhl1b*, and *Fhl1c*) as a result of AS. The results of the present study indicate that in PAH, the use of the most proximal promoter, which transcribes *Fhl1*, increases significantly. The distinct functions of *Fhl1* and *Fhl1b* and their roles in pathogenesis have been reported previously [46]–[48]. In the case of *Rcan1*, 2 splice variants, *Rcan1-1* and *Rcan1-4*, have been reported thus far [49]. The transcription of the splicing variants is specifically regulated by glucocorticoids [50], oxidative stress or Ca^2+^ and calcineurin [51]. Our results indicate that *Rcan1-4* expression increases specifically and strongly in PAH, suggesting the possible mediation of calcineurin pathways in pathogenesis. Among the 2 known isoforms of *Ndrg2* (i.e., *Ndrg2S* and *Ndrg2L*), significant reduction in *Ndrg2L* encoding the long form containing exon 3 is found in PAH [52]. However, the biological significance of the insertion/removal of 14 amino acids (i.e., EAELAARILLDQGQ) encoded by exon 3 has not yet been studied. Multilateral regulation at both quantitative and qualitative levels was also apparent in *Synpo*. The expression level of *Synpo* increased significantly along with the strong inclusion of exon 4 in PAH. However, the isoform that increased significantly in PAH has not been identified yet [53]. *Ttll1* decreased significantly at the quantitative level in PAH. The exclusion of the last exon implies qualitative regulation in *Ttll1*, although UCSC isoforms has not been reported in mice [54]. We found contrasting splicing patterns for *Egfl7* and *Tmpo* depending on the hypertrophic signals. Although there were no significant quantitative changes in these 2 genes, their distinguishable splicing patterns suggest that they are qualitatively regulated by each stimulus.

The differential expression of splicing factors could regulate the exon variants occurring in the hypertrophy models. Our analysis indicates that the expression levels of 2 important splicing factors, *Ptbp1*, which encodes pyrimidine tract-binding protein (PTB), and *A2bp1*, which encodes ataxin 2-binding protein 1 (A2BP1, also known as Fox1), were significantly altered in PAH. Consistent with the previous report regarding post-transcriptional regulation in PAH [55], we also observed significant upregulation of *Ptbp1* (1.9-fold change, *p* = 0.00034) and downregulation of *A2bp1* (0.34-fold change, *p* = 0.0014) in PAH. However, the detailed regulatory mechanisms underlying the exon variants related to the 2 splice factors remains to be elucidated.

### Pathway analysis to distinguish PAH from PHH

The significance of our analysis of the RNA-Seq data to distinguish between the 2 types of hypertrophy using both quantitative and qualitative approaches is clearly shown in Figure 4. Enriched KEGG pathways associated with the DEGs from the 2 types of cardiac hypertrophy were clustered into 5 categories (Figure 4). In general, pathways involved in cardiac muscle contraction and metabolism (Group I) were downregulated, whereas those involved in immune and cell cycle pathways (Group II) were upregulated in PAH. These results are also consistent with those of other microarray studies on PAH [56], [57]. Among them, we are particularly interested in the leukocyte migration pathway, which was significantly activated in PAH. Recently, Damilano et al. [58] reported that PI3Kγ in bone marrow-derived leukocytes might play a critical role in leukocyte infiltration in the myocardium and fibrotic remodeling during PAH. They also suggested the importance of the interplay between the heart and humoral immunity, in that bone marrow-derived leukocytes augment fibrotic remodeling in the heart. In contrast, one of the most striking findings of our study is that the autoimmune-related pathways (Group III) were remarkably suppressed during PHH. Interestingly, genes belonging to the components of antigen presenting systems (e.g., *Tap1*, MHCI and MHCII) were significantly suppressed during exercise. In fact, a recent report showed that immunological stress caused by self-antigens derived from the sudden accumulation of β1-adrenergic receptor can induce the development of PAH in the heart [59]. Suppression of the antigen-presenting system during exercise may prevent pathological progression during cardiac growth. Future studies investigating the interplay between the heart and humoral immunity are necessary. In contrast, pathways involved in signal transduction (Group IV) showed no considerable differences in the DEGs for both hypertrophy models, although AS is more obvious in PHH. These results suggest that exercise provides a more efficient means of signal transduction via AS rather than DEGs. It is interesting to note that the pathways related to heart diseases (Group V) were more sensitive to PAH than PHH with respect to both DEGs and AS, implying that there are significant common pathways for heart diseases.

### Conclusion

RNA-Seq is a revolutionary technology that overcomes the limitations posed by microarray methods. Using RNA-Seq, we found not only DEGs but also different AS patterns during the progression of hypertrophy. These are important issues in the study of cardiac diseases. As exemplified by the unique qualitative changes in the various pathways, some critical domains are likely to be modified via AS without quantitative regulations. Taken together, these findings show the unique transcriptomic signatures of PAH and PHH. In turn, this provides a comprehensive understanding of the pathogenesis of PAH at both quantitative and qualitative levels.

## Materials and Methods

### Ethics Statement

All animal experiments were approved by the Gwangju Institute of Science and Technology Animal Care and Use Committee (Permit number: GIST-2009-14).

### Animal models

8 weeks old male (C57BL/6J) mice (body weight 28–33 g) purchased from SLC Japan were used in all studies.

#### Pathological hypertrophy

Cardiac hypertrophy was induced by TAC operation under anesthesia with intraperitoneal injection of avertin, 2-2-2 tribromoethanol (Sigma, St. Louis, MO) dissolved in tert-amyl alcohol (Sigma, St. Louis, MO). The procedure of operation was followed as previously described [14]. As a control group, sham operation (same procedure except for tying) was done. 1 week after operation, mice were sacrificed, and hearts were removed, and then stored in deep freezer at −80°C before RNA extraction.

#### Physiological hypertrophy

For chronic exercise training, mice swam in water tanks for 4 weeks as described previously [12]. The first day of training consisted of two 10-min sessions separated by at least 4 hrs. The duration of exercise was increased in 10-min increments daily, reaching 90 min, twice daily, by the middle of the second week. This duration of exercise was maintained until 4 weeks. Trained mice were sacrificed and dissected 24 hrs after the last training session to exclude any acute effect of exercise. Dissected hearts were frozen and then stored in deep freezer at −80°C before RNA extraction.

### RNA preparation

Mouse hearts were snap frozen in liquid nitrogen, stores at −80°C, and homogenized in liquid nitrogen using a mortar and pestle. Approximately 450–700 mg of grinded whole mouse heart was used for extraction of total RNA with 1 ml Trizol Reagent® (Invitrogen, Carlsbad, CA) following the manufacturer's instructions. The quality of total RNA was examined by standard RNA chip on by Experion (Bio-Rad Laboratories, Hercules, CA) according to the manufacture's protocol.

### Quantitative real-time PCR (qRT-PCR) and reverse-transcriptional PCR (RT-PCR)

First-strand cDNA was synthesized from 2 µg of total RNA with Random hexamer using Omniscript® reverse transcription (Qiagen, Valencia, CA) according to the manufacturer's instruction. qRT-PCR assays were followed as previously described [60]. Briefly, qRT-PCR assays were performed using SYBR® Premix Ex TaqTM (TaKaRa, Japan) under the following two-step conditions: denaturation at 95°C for 5 seconds; and annealing and extension at 60°C for 40 seconds, for a total of 40 cycles. The 18S transcript was used as an endogenous reference to assess the relative level of mRNA transcript.

RT-PCR assays were performed on a TAKARA thermal cycler TP600 (TaKaRa, Japan) using nTaq-HOT DNA polymerase (Enzynomics, Daejeon, South Korea) under the following 3 step conditions: denaturation at 94°C for 30 s, annealing at 55–60°C for 30 s and extension at 72°C for 40 s with total 30–35 cycles.

### cDNA library preparation for RNA-Seq

Selection of poly(A) mRNA from total RNA was performed using Sera-Mag Magnetic Oligo(dT) Beads (Illumina, San Diego, CA) according to the manufacturer's protocol.

100 ng of mRNA was then used as template for cDNA synthesis. Libraries were prepared according to instructions for mRNA sample preparation kit (Illumina, San Diego, CA). 7 pM of adaptor-ligated DNA was hybridized to the flow cell. DNA clusters were generated using the Illumina cluster station, followed by 38 cycles of sequencing on the Illumina Genome Analyzer, in accordance with the manufacturer's protocols.

### Data processing

The reads obtained by RNA-Seq were compiled using manufacturer-provided pipeline software (Version 1.6). The reads were then aligned onto the mm9 mouse chromosome using Illumina built-in software ELAND2. Only uniquely mapped reads with less than two mismatches were used. The exon coordinates were downloaded from UCSC and analyzed using Illumina supplied software CASAVA.

### Microarray Analysis

Microarray analysis was performed on 100 ng of total RNA samples (DNA link, South Korea) using the Mouse Gene 1.0 ST array (Affymetrix, Foster City, CA). Generation of Labeled cDNA and processing of Mouse Gene 1.0 ST array was performed according to the Affymetrix standard protocol. After staining, intensities were determined with a GeneChip scanner 3000 (Affymetrix, Foster City, CA), controlled by GCOS Affymetrix software.

### Differential Expression Analysis

For the DEG analysis, RPKM values of the genes having ≥2 RPKM were analyzed among sham vs. TAC-operated or sedentary vs. exercise-trained mice by Student's *t*-test (n = 3 for each animal models). For the comparison to microarray, RPKM values of RNA-Seq were converted to logarithmic value of RPKB to help the efficient comparison with the logarithmic value of intensity of Affymetrix chips. For microarray, the expressional intensities across 6 samples were normalized by RMA (Robust Multi-array Average) and logarithmic scaled intensities were further analyzed through Student's *t*-test. In both methods, differentially expressed genes were identified with the significance values of *p*<0.05 and higher than 1.5 fold change.

### AS Analysis

Alternative variants were analyzed based on the reads mapped onto the pre-defined exons based on RefSeq structures. The number of reads for each exon was used for the analyses and the exons with less than 10 RPKM values were discarded to reduce false positives. Fisher's exact test (*p*<0.05) and Bayesian error rate [61] (*e*≤0.1) were used to determine the exon exclusion/inclusion. Exon variants were then compared to knownAlt track of UCSC database. Great enrichment of the specific exons in TAC or exercise samples was considered as exon inclusion, and the significant enrichment of sham-operated or sedentary samples was considered as exon exclusion. For isoform analysis, we applied NEUMA with default parameter for 36-bp single-end reads based on UCSC gene structure [38].

### Enriched Motif Analysis

The enrichment of motifs was analysis for 1,000 bp upstream of genes. For this, the upstream sequences were obtained from UCSC database [62]. Given the upstream sequences, we utilized MEME suite to identify the enriched motifs of which width are ranging 10∼12 bps [18], [63]. Occurrence of a single motif was counted only once for each upstream and reverse complemented motif was not considered. Predicted motifs were further compared against a database of known motifs using TOMTOM [64].

### Enriched KEGG pathway analysis

The enrichment of DEGs for IPA and KEGG pathways was analyzed through Fisher's exact test (*p*<0.05) as follows.
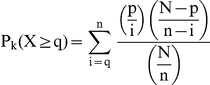
where 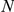 is the total number of genes mapped onto the entire KEGG pathways,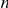 is the number of genes mapped onto the KEGG pathway of interest,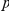 is the total number of DEGs or splicing variants mapped onto the entire KEGG pathways, 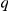 is the number of DEGs or splicing variants mapped onto the KEGG pathway of interest.

### Data accessibility

The RNA-Seq data from this study have been deposited in the EBI ArrayExpress (http://www.ebi.2ac.uk/arrayexpress/) under accession no. E-MTAB-727.

## Supporting Information

Figure S1 **Development of cardiac hypertrophy.** (A) Body weight (BW), heart weight (HW), and heart weight/body weight ratio (HW/BW) at 1 week after TAC operation or at 4 weeks after initiation of exercise training. The average values are shown with standard deviation (N = 3). TAC, transverse aortic constriction. ^**^p<0.01 (TAC vs. Sham). ^††^p<0.01 (Exercise vs. Sedentary). (B) Expression levels of hypertrophic markers in mice models. The differential expressions of the hypertrophic markers were validated using qRT-PCR. All the hypertrophic markers were differentially expressed in TAC-operated mice models whereas only 2 markers (*Nppa* and *Acta1*) were differentially expressed in exercise-trained mice models. Statistically significant at ^*^p<0.05, ^**^p<0.01 and ^***^p<0.001. Bars represent means ± SD.
(TIF)

Figure S2 **Quality assessment of high-throughput data from RNA-Seq.** Reproducibility among the biological replicates derived by (A) RNA-Seq and (B) microarray. To validate experimental reproducibility, linear regression of R-square (R^2^) was calculated among all the replicates derived from RNA-Seq or microarray. The slight expressional differences encountered at the extremely low-level reads could be significantly exaggerated by RPKM normalization. For RNA-Seq, we defined the expression at ≥2 RPKM. (C) Technological comparison of gene expression between RNA-Seq and microarray. The same mRNAs were used for both RNA-Seq and microarray studies. R^2^ was calculated for each replicates. RPKM values of RNA-Seq were transformed into log_2_ scaled RPKB (reads per kilobase per billion mapped reads) to produce a comparable scale. Slightly skewed patterns were observed at low- or high-level expression for all comparisons, suggesting non-linear sensitivity or limited detection capacity of the microarray method at low or high levels of expression.
(TIF)

Figure S3 **Comparison of differentially expressed genes (DEGs) derived from RNA-Seq and microarray.** (A) A dot plot of log fold changes of DEGs obtained by RNA-Seq and microarray is shown. The DEG groups are categorized as: 1) “Common DEGs” showing relatively similar expression patterns between the 2 methods (violet dots) 2) “RNA-Seq specific DEGs” (red dots) and 3) “Microarray specific DEGs” (blue dots). (B) Venn diagram for DEGs derived from RNA-Seq and microarray for PAH (Student t-test, p<0.05, log_1.5_|fold change|≥1). (C–E) Experimental superiority of RNA-Seq over microarray was verified by qRT-PCR. For each series of experiment, the same mRNAs were used for 51 genes tested. Note that the expression profiles obtained by RNA-Seq were significantly more coherent to those obtained by qRT-PCR in PAH (R^2^ = 0.86) (C) and in PHH (R^2^ = 0.89) (D) than those obtained by microarray (R^2^ = 0.69) (E).
(TIF)

Figure S4 **Network of the genes oppositely regulated in PAH and PHH.** (A) Top-scoring network derived from 52 genes oppositely regulated in PAH and PHH. The red color indicates up-regulated genes in PAH. All genes showing increased expression in the network were significantly down-regulated in PHH. (B) The predicted motifs in the 1,000 bp upstream of the 52 oppositely regulated genes. The bottom motifs were predicted by MEME and the motifs above are the consensus ones for MAZ. Degree of expression of *Maz* is shown in the order of Sham (Sh), TAC (T), Se (Sedentary) and E (exercise).
(TIF)

Figure S5 **Summary data of the alternative splicing patterns for PAH and PHH.** (A) Summary of the method to detect alternative exons by RNA-Seq. Significant exon variants were identified from 182,627 exons in the UCSC database by filtering with Bayesian probability (e≤0.1), Fisher's exact test (p<0.05), and knownAlt tracks from the UCSC database. (B, C) Pie charts representing types of exon variants for (B) PAH and (C) PHH. The types of alternative splicing for the exon variants in hypertrophy were examined on the basis of known UCSC ‘knownAlt’ tracks. The most abundant AS types were grouped as alternative promoters, cassette exons and bleeding exons for both types of hypertrophy.
(TIF)

Table S1 **Summary of read number.** Given 13∼20 million reads, 7∼11 million reads were mapped to known genes in mm9 mouse genomes. Only the reads that have ≥2 RPKM were selected for “expressed genes for each models.
(DOC)

Table S2 **The primer sets for amplification of exon variants shown in** **Figure 4** **.** F and R represent forward and reverse, respectively.
(DOC)
