# Supplementary figures and images for: Deep RNA Sequencing Reveals Novel Cardiac Transcriptomic Signatures for Physiological and Pathological Hypertrophy
Source: PLoS One. 2012 Apr 16;7(4):e35552. doi: 10.1371/journal.pone.0035552 (PMC3327670; doi:10.1371/journal.pone.0035552)

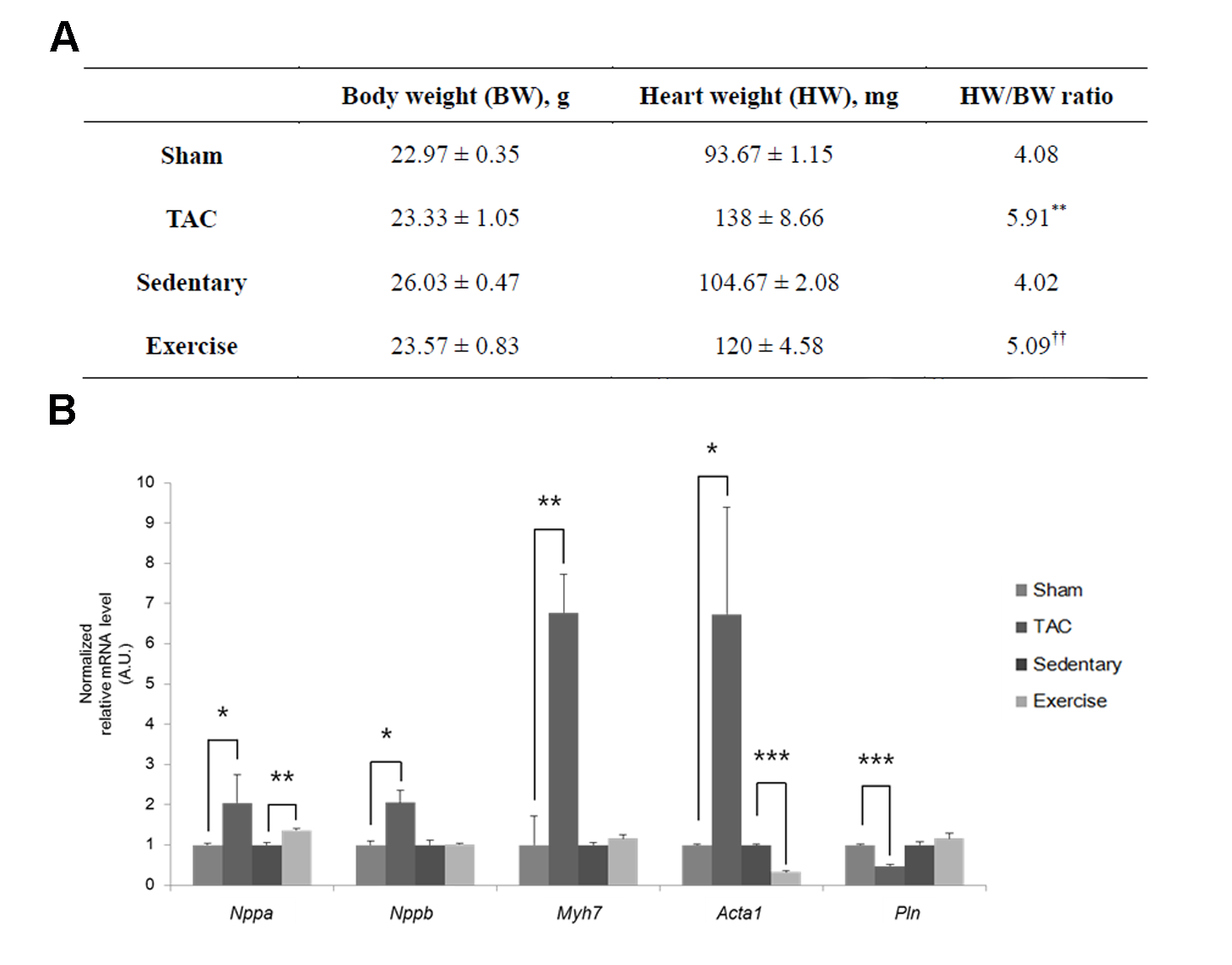

Supplement: Figure S1 — Development of cardiac hypertrophy. (A) Body weight (BW), heart weight (HW), and heart weight/body weight ratio (HW/BW) at 1 week after TAC operation or at 4 weeks after initiation of exercise training. The average values are shown with standard deviation (N = 3). TAC, transverse aortic constriction. **p<0.01 (TAC vs. Sham). ††p<0.01 (Exercise vs. Sedentary). (B) Expression levels of hypertrophic markers in mice models. The differential expressions of the hypertrophic markers were validated using qRT-PCR. All the hypertrophic markers were differentially expressed in TAC-operated mice models whereas only 2 markers (Nppa and Acta1) were differentially expressed in exercise-trained mice models. Statistically significant at *p<0.05, **p<0.01 and ***p<0.001. Bars represent means ± SD. (TIF) [file pone.0035552.s001.tif]

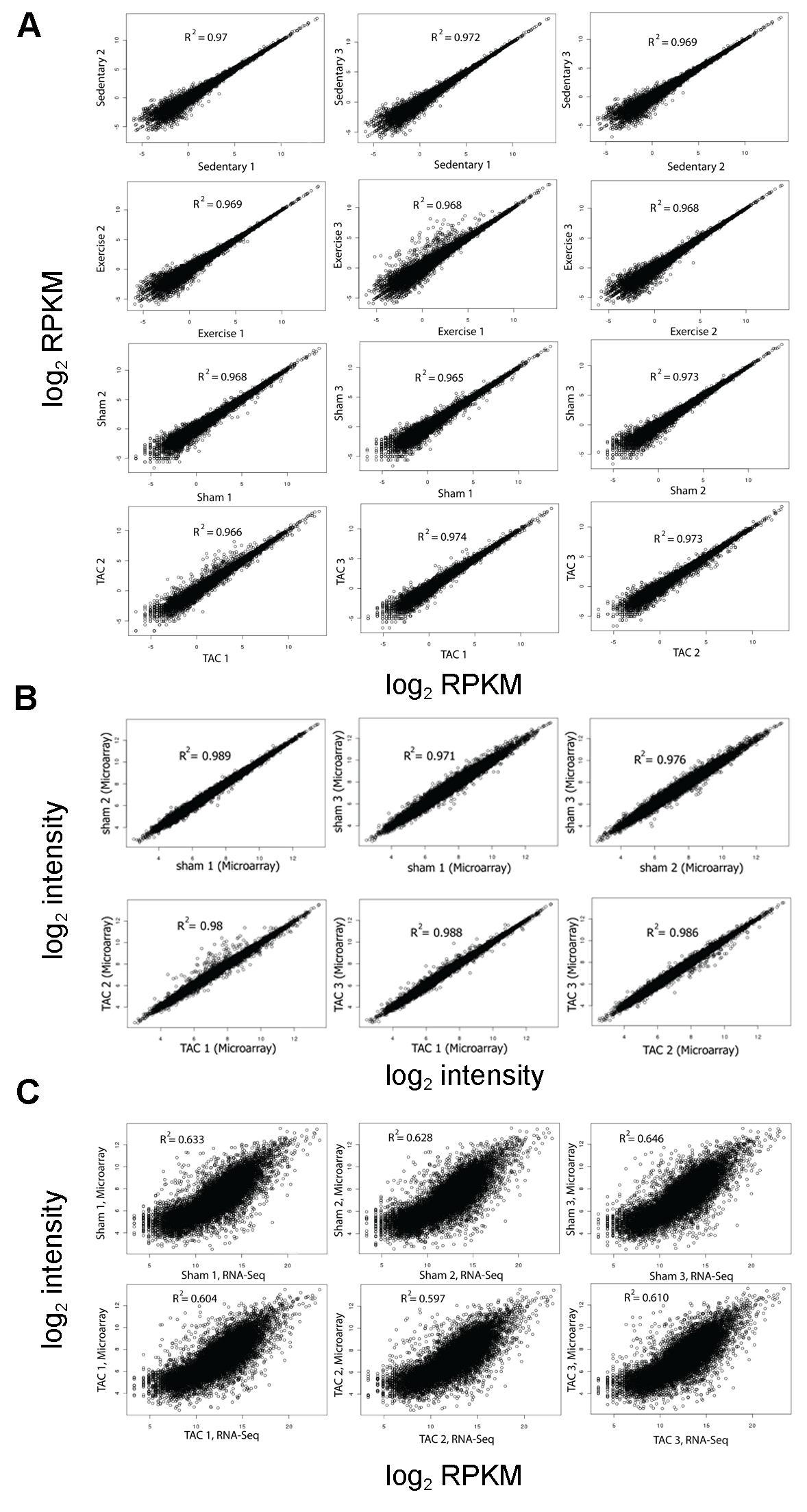

Supplement: Figure S2 — Quality assessment of high-throughput data from RNA-Seq. Reproducibility among the biological replicates derived by (A) RNA-Seq and (B) microarray. To validate experimental reproducibility, linear regression of R-square (R2) was calculated among all the replicates derived from RNA-Seq or microarray. The slight expressional differences encountered at the extremely low-level reads could be significantly exaggerated by RPKM normalization. For RNA-Seq, we defined the expression at ≥2 RPKM. (C) Technological comparison of gene expression between RNA-Seq and microarray. The same mRNAs were used for both RNA-Seq and microarray studies. R2 was calculated for each replicates. RPKM values of RNA-Seq were transformed into log2 scaled RPKB (reads per kilobase per billion mapped reads) to produce a comparable scale. Slightly skewed patterns were observed at low- or high-level expression for all comparisons, suggesting non-linear sensitivity or limited detection capacity of the microarray method at low or high levels of expression. (TIF) [file pone.0035552.s002.tif]

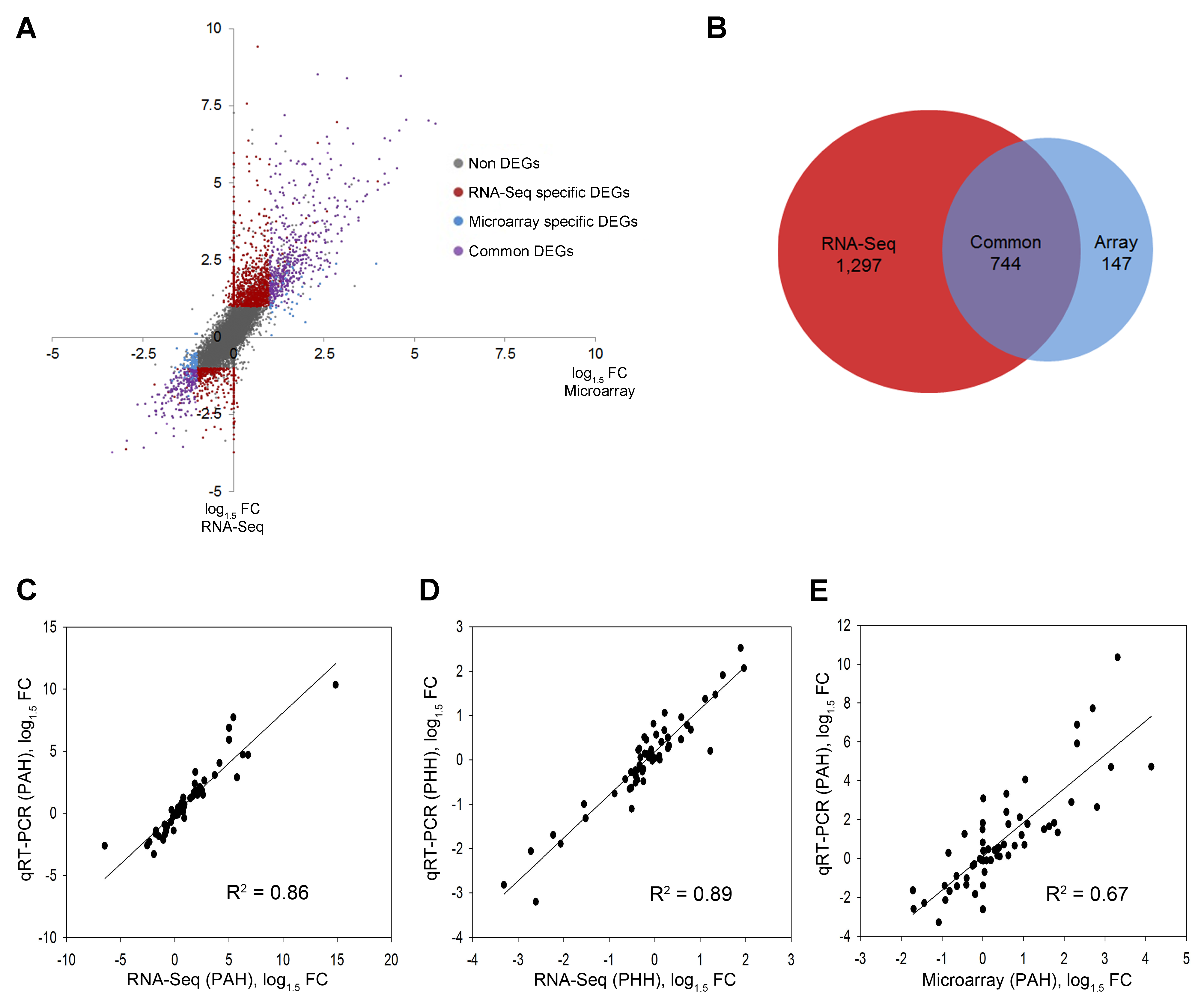

Supplement: Figure S3 — Comparison of differentially expressed genes (DEGs) derived from RNA-Seq and microarray. (A) A dot plot of log fold changes of DEGs obtained by RNA-Seq and microarray is shown. The DEG groups are categorized as: 1) “Common DEGs” showing relatively similar expression patterns between the 2 methods (violet dots) 2) “RNA-Seq specific DEGs” (red dots) and 3) “Microarray specific DEGs” (blue dots). (B) Venn diagram for DEGs derived from RNA-Seq and microarray for PAH (Student t-test, p<0.05, log1.5|fold change|≥1). (C–E) Experimental superiority of RNA-Seq over microarray was verified by qRT-PCR. For each series of experiment, the same mRNAs were used for 51 genes tested. Note that the expression profiles obtained by RNA-Seq were significantly more coherent to those obtained by qRT-PCR in PAH (R2 = 0.86) (C) and in PHH (R2 = 0.89) (D) than those obtained by microarray (R2 = 0.69) (E). (TIF) [file pone.0035552.s003.tif]

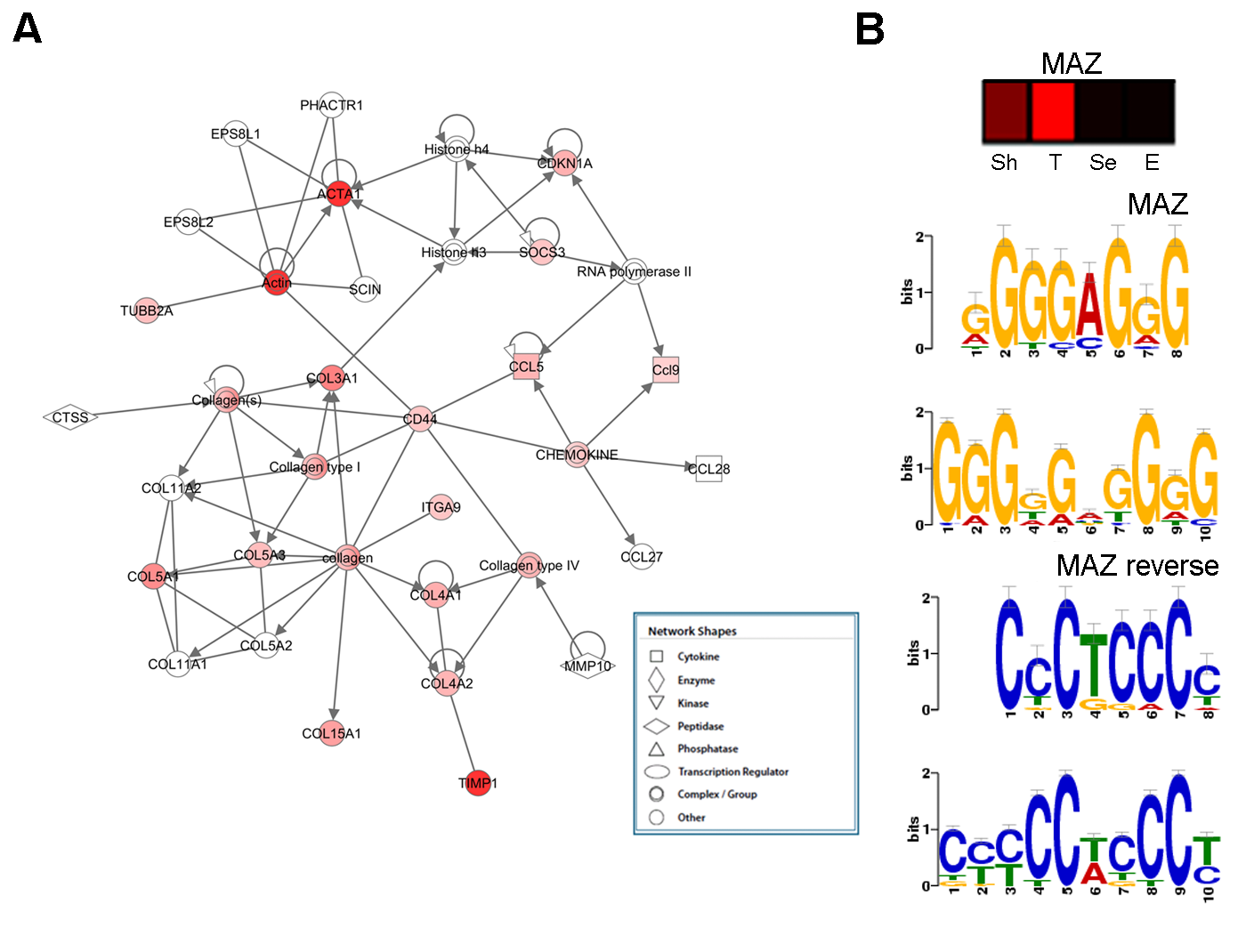

Supplement: Figure S4 — Network of the genes oppositely regulated in PAH and PHH. (A) Top-scoring network derived from 52 genes oppositely regulated in PAH and PHH. The red color indicates up-regulated genes in PAH. All genes showing increased expression in the network were significantly down-regulated in PHH. (B) The predicted motifs in the 1,000 bp upstream of the 52 oppositely regulated genes. The bottom motifs were predicted by MEME and the motifs above are the consensus ones for MAZ. Degree of expression of Maz is shown in the order of Sham (Sh), TAC (T), Se (Sedentary) and E (exercise). (TIF) [file pone.0035552.s004.tif]

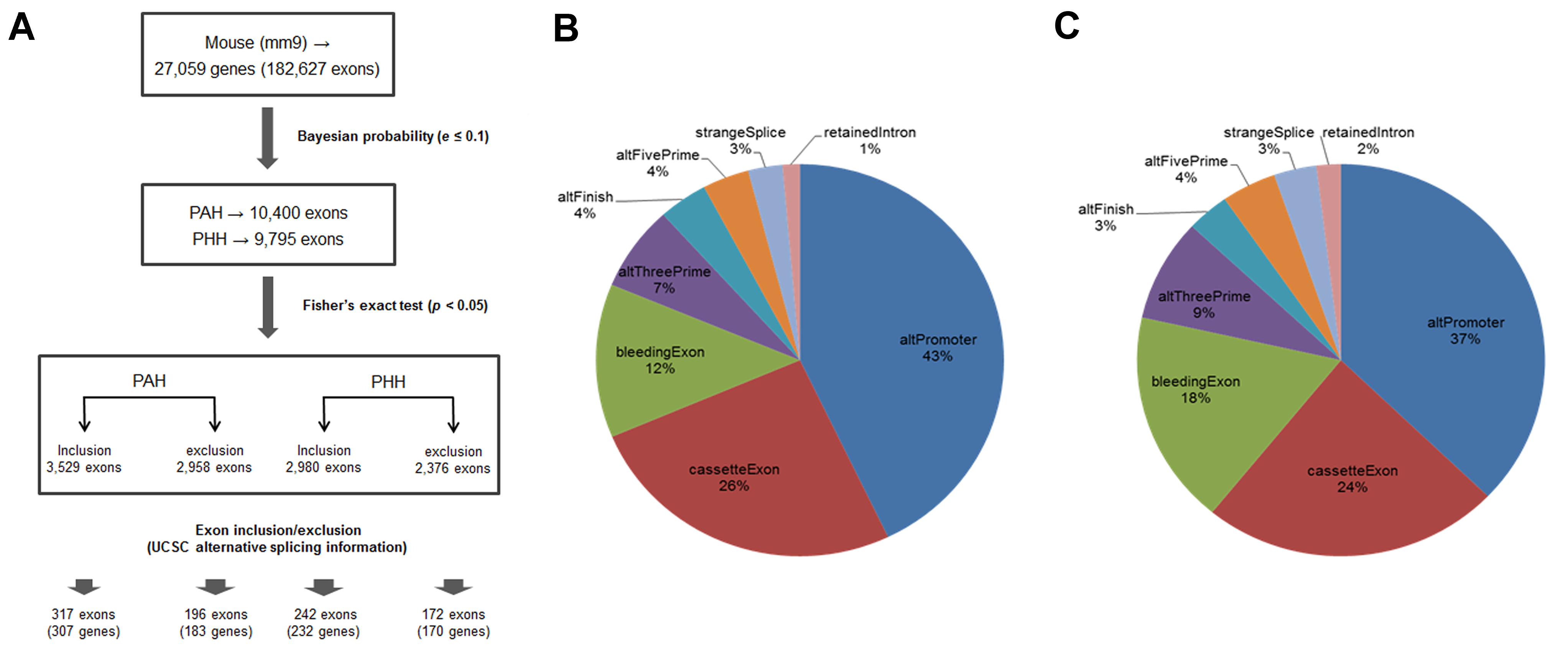

Supplement: Figure S5 — Summary data of the alternative splicing patterns for PAH and PHH. (A) Summary of the method to detect alternative exons by RNA-Seq. Significant exon variants were identified from 182,627 exons in the UCSC database by filtering with Bayesian probability (e≤0.1), Fisher's exact test (p<0.05), and knownAlt tracks from the UCSC database. (B, C) Pie charts representing types of exon variants for (B) PAH and (C) PHH. The types of alternative splicing for the exon variants in hypertrophy were examined on the basis of known UCSC ‘knownAlt’ tracks. The most abundant AS types were grouped as alternative promoters, cassette exons and bleeding exons for both types of hypertrophy. (TIF) [file pone.0035552.s005.tif]
