# Supplementary material for: Deep RNA Sequencing Reveals Novel Cardiac Transcriptomic Signatures for Physiological and Pathological Hypertrophy
Source: PLoS One. 2012 Apr 16;7(4):e35552. doi: 10.1371/journal.pone.0035552 (PMC3327670; doi:10.1371/journal.pone.0035552)
Supplement: Table S1 — Summary of read number. Given 13∼20 million reads, 7∼11 million reads were mapped to known genes in mm9 mouse genomes. Only the reads that have ≥2 RPKM were selected for “expressed genes for each models. (DOC) [file pone.0035552.s006.doc]

|  | **Sham** | | | **TAC** | | | **Sedentary** | | | **Exercise** | | |
| --- | --- | --- | --- | --- | --- | --- | --- | --- | --- | --- | --- | --- |
| **Reads Category** | Sham 1 | Sham 2 | Sham 3 | TAC 1 | TAC 2 | TAC 3 | Sedentary 1 | Sedentary 2 | Sedentary 3 | Exercise 1 | Exercise 2 | Exercise 3 |
| **Total Reads** | 19,721,864 | 20,440,207 | 20,491,296 | 20,108,180 | 19,730,589 | 19,504,520 | 14,089,543 | 14,689,691 | 14,374,893 | 14,473,848 | 13,789,493 | 13,639,331 |
| **Mapped Reads^a^ (mm9 mouse genome)** | 12,076,604 | 12,608,889 | 12,438,094 | 13,456,173 | 12,830,171 | 12,927,635 | 8,409,552 | 8,508,886 | 8,631,502 | 8,213,690 | 7,792,221 | 8,137,049 |
| **Mapped Reads^b^ (known gene collection)** | 10,458,455 | 10,922,911 | 10,601,413 | 11,858,319 | 11,298,956 | 11,351,818 | 7,099,363 | 7,159,197 | 7,326,281 | 7,008,951 | 6,616,302 | 6,939,598 |
| **Not Mapped Reads (known gene collection)** | 1,618,149 | 1,685,978 | 1,836,681 | 1,597,854 | 1,531,215 | 1,575,817 | 1,310,189 | 1,349,689 | 1,305,221 | 1,204,739 | 1,175,919 | 1,197,451 |
| **Number of Gene (Read ≥ 1)** | 16,922 | 17,020 | 17,285 | 17,347 | 17,220 | 17,243 | 16,666 | 16,637 | 16,725 | 16,324 | 16,407 | 16,297 |
| **Number of Gene (RPKM ≥ 2)** | 9,954 | 9,963 | 9,896 | 10,352 | 10,309 | 10,373 | 10,095 | 10,070 | 10,090 | 9,960 | 9,996 | 10,009 |

^a^number of reads mapped to the mouse genome (mm9, NCBI build 37) ^b^number of reads matched to the RefSeq sequence.
