# Supplementary material for: Deep RNA Sequencing Reveals Novel Cardiac Transcriptomic Signatures for Physiological and Pathological Hypertrophy
Source: PLoS One. 2012 Apr 16;7(4):e35552. doi: 10.1371/journal.pone.0035552 (PMC3327670; doi:10.1371/journal.pone.0035552)
Supplement: Supplementary file 3 [file pone.0035552.s007.doc]

Name of primer	Sequence		Ndrg2-F 	TCAAAGGCAAGTGAAGGTGG		Ndrg2-R	CGAGCCATAAGGTGTCTCCA		Dscr1-F1	GACTGCGAGATGGAGGAGGT		Dscr1-F2	CTCCCTGATTGCTTGTGTGG		Dscr1-R	TCATTTCCTTCCCCAGGAAC		Efgl7-F1	TGAGGGGTCCAAGGAGAGTC		Efgl7-F2	GGCCACAAAAAGAAGAAGGC		Efgl7-R	ACACGCTGCACAAAGGTCTC		Ttll1-F1	CTG GGCGGTTTCTAAGCTCT		Ttll1-R1	CGTCTCTCACATCCAGCACC		Ttll1-F2	AGCACAGGGAAAAGGCATCT		Ttll1-R2	ACTTCACTGTGCAGAAGCGG		Fhl1-F2	CCTCCAGCTATAAGGTGGGC		Fhl1-R	GAAGCAGTTGTCGTGCCAGT		Fhl1-F1	AGGGAAGACAGGTCTGGGTG		Tmpo-F2	CAGCGTTTGTTCATGGAAGG		Tmpo-R2	CAATCTGGATGGATGGGACA		Tmpo-F1	ATCTTCAACAGGCGGACCTC		Tmpo-R1	TGG CCACTAAGGATTCGTTG		Synpo-F1	AACCTGGGTGGGAAGTCAAG		Synpo-R1	AGCTCTTCTTCCCCCAGTCA		Synpo-F2	TGGCTTCTCGGGTTAGGTCT		Synpo-R2	TCCGCTTCTCATCAGCTGTC		Cxxc5-F1	GCCAGAAGAGGAGAGCCTCA		Cxxc5-F2	CCTACCCTGACTGAGGCTCC 		Cxxc5-R	ACTGCTGCTGCTACTGCCAC
